# Impact of external sources of infection on the dynamics of bovine tuberculosis in modelled badger populations

**DOI:** 10.1186/1746-6148-8-92

**Published:** 2012-06-27

**Authors:** Joanne L Hardstaff, Mark T Bulling, Glenn Marion, Michael R Hutchings, Piran C L White

**Affiliations:** 1 Environment Department, University of York, York, YO10 5DD, UK; 2 Biomathematics and Statistics Scotland, James Clerk Maxwell Building, Kings Buildings, Edinburgh, EH9 3HH, UK; 3 Disease Systems, Scottish Agricultural College, West Mains Road, Edinburgh, EH9 3JG, UK; 4 Biological Sciences, University of Derby, Kedleston Road, Derby, DE22 1 GB, UK

**Keywords:** Cattle, Disease, Host community, Model, Threshold, Perturbation

## Abstract

**Background:**

The persistence of bovine TB (bTB) in various countries throughout the world is enhanced by the existence of wildlife hosts for the infection. In Britain and Ireland, the principal wildlife host for bTB is the badger (*Meles meles*). The objective of our study was to examine the dynamics of bTB in badgers in relation to both badger-derived infection from within the population and externally-derived, trickle-type, infection, such as could occur from other species or environmental sources, using a spatial stochastic simulation model.

**Results:**

The presence of external sources of infection can increase mean prevalence and reduce the threshold group size for disease persistence. Above the threshold equilibrium group size of 6–8 individuals predicted by the model for bTB persistence in badgers based on internal infection alone, external sources of infection have relatively little impact on the persistence or level of disease. However, within a critical range of group sizes just below this threshold level, external infection becomes much more important in determining disease dynamics. Within this critical range, external infection increases the ratio of intra- to inter-group infections due to the greater probability of external infections entering fully-susceptible groups. The effect is to enable bTB persistence and increase bTB prevalence in badger populations which would not be able to maintain bTB based on internal infection alone.

**Conclusions:**

External sources of bTB infection can contribute to the persistence of bTB in badger populations. In high-density badger populations, internal badger-derived infections occur at a sufficient rate that the additional effect of external sources in exacerbating disease is minimal. However, in lower-density populations, external sources of infection are much more important in enhancing bTB prevalence and persistence. In such circumstances, it is particularly important that control strategies to reduce bTB in badgers include efforts to minimise such external sources of infection.

## Background

A common factor complicating the control of livestock diseases is that many such diseases are capable of infecting multiple wildlife hosts [1-3]. One of the most persistent diseases with a wide host range is bovine tuberculosis (bTB), which is caused by the bacterium *Mycobacterium bovis* and is a major concern to the livestock industry in many countries, including the UK [4]. The existence of wildlife hosts has reduced the success of bTB control programmes in certain countries. In Europe, bTB occurs in a wide range of species [3,5]. Commonly affected wildlife species are wild boar (*Sus scrofa*), red deer (*Cervus elaphus*), fallow deer (*Dama dama*), roe deer (*Capreolus capreolus*), badgers (*Meles meles*) and red foxes (*Vulpes vulpes*) [5].

In Britain and Ireland, badgers are particularly important in enabling the persistence of bTB in cattle [6]. Badger populations are structured socially and spatially in a territorial system, but the size of groups varies considerably, largely as a consequence of habitat quality [7,8]. Group sizes in Britain tend to be higher than in continental Europe, particularly in local areas with a history of persistent bTB in cattle. For example, group sizes in south-west Britain, a region of high bTB prevalence, range between 3–10 individuals [9], with exceptional group sizes up to 27 individuals [10]. These compare with group sizes of 2–7 in Poland and Spain [11,12] and 6–8 in Portugal [13]. Studies using epidemiological models suggest a threshold group size of around 6–8 individuals is required to enable bTB to persist in badger populations in Britain in the absence of external infection [14-16]. However, bTB can persist where badger group sizes are below such levels, as is the case in Ireland where the mean badger group size has been estimated to be 3.9 (3.41-4.45) badgers per sett [17] with a different study observing a range of 1–14 badgers per sett [18]. The persistence of disease at lower population levels may be enabled by variations in the social structure of the badger host population in different situations [19]. However, it may also be enabled by the existence of infection within a wider host community, including livestock, which through either direct or environmental transmission of the infection to badgers may effectively lower the threshold population density required for disease persistence. Such external sources of infection, originating from outside the focal host species, are thought to be one of the mechanisms underlying the persistence of canine distemper in Yellowstone National Park [20] and enables the disease to be transmitted among neighbouring populations, such as from grasshopper mice (*Onychomys leucogaster*) to prairie dog (*Cynomys ludovicianus*) populations [21]. It is also thought that external, but as yet unexplained, sources of infection are responsible for the high levels of bTB in the New Zealand feral pig population [22]. Understanding the impact of sources external to the badger population on the dynamics and persistence of bTB is therefore important for the development of more effective and targeted bTB control strategies.

In this paper, we use a spatial, stochastic simulation model to examine the relative effect of badger group size, internal (badger intra- and inter-group) infection rates, and infection from external sources (*i.e.* non-badger) on the prevalence and persistence of bTB within badger populations across a wide range of equilibrium group sizes found in Europe. We use two different modelling scenarios. Scenario 1 considers solely badger-derived infection and scenario 2 considers both badger-derived and trickle-type infection from external sources of infection.

## Results

### Sensitivity analysis

This section summarises the full sensitivity analysis, which is presented in the Additional file 1. Colonisation and dispersal were the most importance influences on mean group size in the absence of external infection, accounting for 21% and 15% respectively of variations in group size at an equilibrium group size of 8. Colonisation and dispersal by females had a slightly greater impact than colonisation and dispersal by males, especially for smaller group sizes, probably because of the more direct impact that females have on the group’s reproductive potential. As equilibrium group size increases, a group is more likely to contain more than a critical number of females, and hence the relative importance of the female parameters in influencing group size and prevalence declines. As rates of external infection increase, colonisation becomes less important in influencing group size. For example, the influence of adult female colonisation on variation in group size for an equilibrium group size of 4 decreased from 49% to 30% as the rate of external infection increased from 0.0001 to 0.01. However, as rates of external infection increased, disease-related parameters such as intra-group transmission and the rate at which badgers changed between infectious and latent states, assumed greater importance in affecting group size. For example, the influence of the transfer of females from a latent to infectious state at an equilibrium group size of 4 increased from 0.06% at an external infection probability of 0.0001 to 30% at an external infection probability of 0.01.

Intra-group transmission was the dominant disease-related parameter overall in terms of its effect on prevalence, accounting for between 30% and 78% of the variation in prevalence for equilibrium group sizes of 4, 8 and 12 at all external infection probabilities. Its influence was greatest for the higher group sizes (8 and 12). The importance of intra-group infection reflects the spatio-temporally persistent nature of bTB in badger populations, and is representative of a disease that is generally maintained through interactions within rather than between groups. At lower group sizes, at or just below the threshold population density for disease persistence, the parameters of infection itself, such as disease-induced mortality and the rates of transfer between different infectious states, became more important in influencing prevalence. In the absence of disease, the badger population persisted at all group sizes, although the inclusion of disease at the smallest equilibrium group size of 2 did contribute to the extinction of the badger population in up to 8% of scenario simulations at this group size. Populations did not go extinct at any higher group sizes under either scenario.

### Badger-derived infection only (scenario 1)

**Figure 1:**
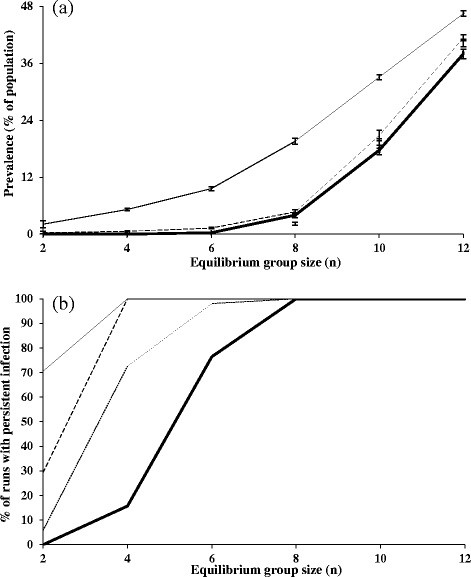
**Relationships of (a) mean prevalence and (b) percentage of model runs in which disease has persisted as a function of equilibrium group size.** The thick line shows badger-derived infection only and the thin black lines show combined badger-derived and external sources of infection (scenario 2) with probabilities of 0.0001 (thin dotted line), 0.001 (thin dashed line) and 0.01 (thin solid line). Vertical bars denote 95% confidence intervals.

With infection derived solely from within the badger population, higher prevalences were obtained with higher equilibrium group sizes (Figure 1a), and increased non-linearly with an increase in equilibrium group size, after equilibrium group sizes reached 6. There was a clear threshold group size of around 6–8 individuals required for the disease to persist in the population (Figure 1b). At an equilibrium group size of 8 individuals, the disease persisted in all simulation runs (Figure 1b) at a mean prevalence of 5%, and for an equilibrium group size of 12, the mean prevalence was 36%.

**Figure 2:**
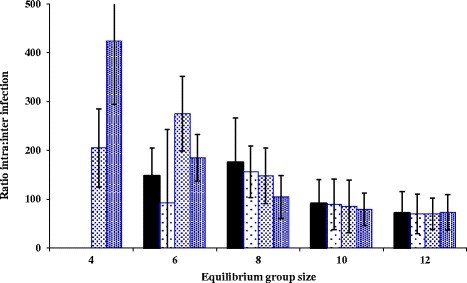
**The ratio of intra to inter-group infection rates for different sources of infection where disease persisted.** The graph shows badger-derived infection (scenario 1; solid bars) compared with badger-derived and externally-derived infection (scenario 2; dotted bars). For scenario 2, the low density-dotted bars represent an externally-derived transmission probability of 0.0001, the medium density-dotted bars represent a probability of 0.001 and the high density-dotted bars represent a probability of 0.01. 95% confidence intervals are also shown. For a group size of 4, infection did not persist unless the probability of externally-derived infection was at least 0.001.

When disease became established in the population, at an equilibrium group size of 6 or above, intra-group transmission events dominated the dynamics of disease (Figure 2). The relative importance of intra-group to inter-group transmission events declined slowly as group size increased.

### Badger-derived and external infections combined (scenario 2)

The lowest probability of external infection (0.0001) had a negligible effect on the mean prevalence of the disease compared with badger-derived infection alone (Figure 1a). A marked increase in mean prevalence was only obtained with the highest probability of external infection (0.01). For an equilibrium group size of 8, the mean prevalence was 19% at an external infection probability of 0.01, compared with between 2% and 4% for lower external infection probabilities of 0.001 and 0.0001. However, external infection at all levels had a much greater impact on disease persistence. Even the lowest probability of external infection (0.0001) enabled the disease to persist much more readily in populations with a mean group size below the badger-only infection threshold density (Figure 1b). With higher probabilities of external infection (0.001 and 0.01), this effect became more pronounced. The relative impact of external infection in increasing disease persistence and prevalence was greatest at or around, and particularly just below, the equilibrium group size. At an equilibrium group size of 4 individuals, disease persisted in only 15% of simulations when infection was solely badger-derived. However, when badger-derived infection was supplemented by external infection, disease persisted in 75% of simulations at the lowest external infection probability (0.0001), and persisted in all simulations at the intermediate (0.001) and highest probabilities (0.01) of external infection (Figure 1b).

When disease became established in the population as a consequence of both badger-derived and external infection, the relative pattern of intra- and inter-group transmission was similar to that obtained with badger-derived infection alone, with the relative importance of intra-group to inter-group transmission events declining as group size increased beyond the threshold group size of 6. However, with the two highest probabilities of external infection (0.001 and 0.01), the relationship was shifted to the left (Figure 2) with a significant number of intra-group transmission events now occurring at equilibrium group size of 4.

## Discussion

Our results are consistent with those from other computer models that support the existence of a threshold group size for the persistence of bTB in badger populations at around 6–8 adults per group [14-16]. The general increases in prevalence with group size, albeit it in a non-linear pattern, also support the broader literature on the dynamics of disease in a range social host species [23]. However, recent empirical work has found a negative relationship between bTB prevalence and badger abundance, with prevalence highest in smaller social groups and at lower population densities [24]. The most plausible hypothesis to explain this finding is that badger contact behaviour changes according to group size, and greater mobility of badgers between groups at lower population densities may result in a higher proportion of contacts leading to disease transmission [24]. However, there are no behavioural data available from free-living badger populations at low densities to test this hypothesis or to re-parameterise the model. In the absence of such data, the model assumed that individual badgers at all densities exhibited the behaviour patterns recorded in a moderate-density population [25], and this assumption probably explains the predicted increases in prevalence with group size in the model. A relatively higher frequency of contacts between badgers at lower densities would have the effect of lowering the threshold for disease persistence in much the same way as increased external transmission does, and thus the predictions of the model for lower social group sizes should be treated with caution, and considered as lower bounds in terms of disease persistence and prevalence.

The addition of external trickle-type infection in the model decreased the threshold group size for bTB persistence [26], allowing for persistence in smaller group sizes, and raised the prevalence in larger group sizes. It enabled an increase in prevalence to occur in already infected populations that consisted of larger group sizes. The driver for this was an increase in intra-group transmission rates. However, this effect was not consistent across all group sizes, and relatively greater impacts were achieved at lower group sizes, particularly just below the threshold level. At these lower group sizes, there is a more heterogeneous distribution of infection across groups, and hence it is more likely that external infection will result in disease being introduced into fully susceptible groups that do not have contiguous borders with infected groups. Following introduction of disease into susceptible groups, the relatively higher rates of intra-group transmission lead to higher levels of disease. These results suggest that only relatively minor changes in intra-group contact behaviour at lower densities may be sufficient to have a significant impact on the persistence of bTB in lower-density populations. With high levels of external infection, bTB is thus able to persist in smaller badger social groups commonly found within Europe [27], as well as in Ireland [18]. In these situations, badger populations are likely to be fragmented, and external infections may provide a means for infection to reach those groups which would otherwise have been relatively isolated from dispersing individuals from other sub-populations.

External infections of badger populations may arise from livestock, other wildlife or environmental sources. For example, deer can shed *M. bovis* in faecal matter and nasal mucous [28] which can contaminate the environment, and cattle can shed *M. bovis* through nasal mucous [29] that can contaminate water sources [30]. In larger badger group sizes, for example above 10–12, as are found in parts of south-west England where bTB is believed to be endemic in the badger population [31], external infection is not required for disease persistence. Nevertheless, even in these situations, significant external infection can cause increases in prevalence when there is endemic disease. Where these external sources of infection include maintenance hosts, disease control strategies should include management of these other potential sources of disease. However, effective control of bTB in the badger population may also help to reduce the significance of these external sources, especially if the other species are acting solely as spillover hosts [32]. With larger group sizes, even occasional infectious disease contact can cause low levels of disease to persist, thereby complicating control measures.

Studies on bTB in other wildlife in south-west England have found several other wildlife species to be infected with bTB, for example various rodent species, foxes, red deer, roe deer, fallow deer and muntjac (*Muntiacus reevesi*) [33]. However, only some of the affected species excrete bTB and are therefore capable of contributing to a pool of external infection. Of the affected species, only fallow deer and muntjac, at densities of over 56 per km^2^ and 47 per km^2^ respectively, are likely to act as maintenance hosts [32]. Spillover host status may be provided by roe deer, red deer, muntjac and fallow deer at lower densities [32,34]. However, the capacity of these species to maintain the disease as part of a multi-species host community has yet to be explored, and it may provide further opportunities for external infection to the badger population, as well being able to act as a spillover for bTB from badgers. Moreover, the presence of multispecies hosts of disease increases the possibility of contact across a wider variety of habitats due to a range of differences between species including foraging and dispersal distances. For example, the foraging distance of a badger may only reach 250 m whereas the foraging distance of a roe deer doe may be more than 1800 m [35,36].

It is likely that a low level of seeding from external sources, for example external seeding from infected livestock and/or from infected environmental sources such as water, food and soil [37] occurs in many areas where bTB persists in wildlife populations around the world. In Northern Michigan, a survey of wildlife for *M.bovis* infection discovered infected opossums (*Didelphis virginianus*) and a grey fox (*Urocyon cinereoargenteus*) which had been located on bTB-depopulated cattle farms [38]. *M. bovis* has been recovered from soil samples [39], badger setts and badger latrines [40] in areas with infected badgers, and has shown to be able to survive for long periods of time within soil [41] and on the forest floor in areas of high possum (*Trichosurus vulpecula*) density [42]. Our analysis of external infection of bTB in badgers has shown that low levels of external infection alone are relatively unimportant compared with disease processes internal to the host population in determining the persistence of bTB in badgers above the threshold group size. However, at lower group sizes, as occur throughout many areas where bTB persists in cattle, external sources of infection are likely to assume a much greater significance. This may account for bTB infection in badgers in Switzerland, France and Spain [27,43]. For example, bTB persists in badger populations in Ireland despite an average group size of 3.9 badgers [17]. All else being equal, a greater level of external infection would be required to maintain bTB in badgers in Ireland, because of the smaller badger group sizes, than is the case in Britain, where group sizes are frequently well above the threshold level predicted by the model. However, our results suggest that the Irish situation is also more sensitive to the effects of external infection than that in Britain, since the group size in Ireland is within a critical range just below the threshold for disease persistence, where the potential role of any external sources is magnified. In Ireland, bTB persistence is therefore likely to be a consequence of a combination of badger-derived infection, infection from livestock and from other environmental sources [44,45], as well as possible differences in badger behaviour at lower densities [24]. In many parts of Britain, especially where badger densities are higher, bTB is likely to be maintained simply through badger-derived infection.

The control of bTB has been based historically on the testing and slaughter of infected cattle, or taken a dual approach, combining a test-and-slaughter policy with wildlife culling [46,47]. Culling of badgers may result in more dispersal of individuals, with the potential to lead to a perturbation effect (higher bTB as a consequence of disruption to the badger social system due to badger culling [48,49]). However, the extent of the any perturbation effect is likely to depend on both the extent of behavioural change in the badger population relative to the previous situation, as well as on the relative dependence of the disease system on badger-derived infection compared with cattle-derived infection. Perturbation is more likely to occur where bTB persistence is predominantly badger-derived, and where the effects of culling are to cause significant changes in behavioural patterns.

Our work suggests that the effectiveness of bTB control through badger culling is therefore dependent on the interplay between badger population density and group size (which determine social behaviour and the likely extent of any perturbation effect), and the presence and significance of external sources of infection. As badger populations are decreased by culling, especially where initially high-density populations are reduced significantly, disease may transiently increase due to a perturbation effect. Where a disease problem is still present in the cattle, disease may continue to persist, or even resurge, due to the role of cattle-derived infection in the system [50,51], and this may be exacerbated by longer-term changes in the behaviour of the badger population. Any culling-based strategy to reduce bTB in badgers therefore needs to aim to reduce badger populations to not only below the level at which perturbation effects on disease are most pronounced, but also below the critical range where the disease in badgers can be maintained by trickle-type infection from other sources in the ecosystem.

## Conclusions

Our analysis has shown that additional sources of infection, such as other infected bTB-excreting wildlife, environmental contamination, and cattle failing to test positive and being removed from a farm due to imperfect test sensitivity [52] will reduce the effectiveness of disease management. In our model, external trickle-type infection only occurred during the summer season. Whilst the overall amount of external infection in the system is unknown, it may also occur at other times of the year, especially where badgers enter farm buildings frequently [53]. The addition of external infection at other times of the year would further enhance its role in enabling bTB persistence in badger populations at sub-threshold densities. This highlights the need for integrated strategies for bTB management, addressing both the livestock-wildlife host system and other environmental sources of infection, to reduce the risk of bTB in badger populations.

## Methods

### Model structure and parameters

We used a spatial simulation model following the structure of that in White and Harris [15]. This model shares the same general form as the badger-bTB model produced by the Food and Environment Research Agency [14,16,54] and used by the Department of Environment, Food and Rural Affairs (Defra) to inform the management of bTB in badgers in England. The models have produced similar predictions regarding the ecology and management of bTB in badger populations, including the identification of a threshold badger group size of around 6–8 individuals for disease persistence.

**Figure 3:**
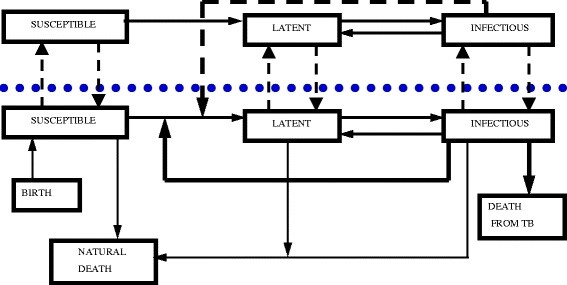
**Structure of the model showing disease dynamics.** The dotted line indicates the separation between adjacent cells of the model which represent the boundary between two adjacent badger territories to demonstrate movement of individuals of different infectious states between groups. The additional compartments of birth, natural death and mortality from bTB are also included below the line to show how individuals enter and leave the population, and these processes are common to all cells in the model. The short hashed lines represent disease transmission events, thick continuous lines represent state transitions colonisation or dispersal events and thin continuous lines represent demographic processes.

The White and Harris model was adapted to allow continued infection from external sources and enable different forms of disease control (Figure 3). Space in the model was represented by a grid of square cells (12 x 12), with each cell representing a badger territory. Within a territory, the numbers of individuals in each age (cub, yearling, adult), sex (male, female) and disease state (susceptible, latent, infectious) category were tracked through time. The model was stochastic, with progression of disease between susceptible, latent and infectious states based on probabilities derived from the literature (Table 1). For each potential transition between disease states, a random number between 0 and 1 was generated and the transition occurred if that number was less than or equal to the specific probability. Transmission of infection could occur within territories (intra-group transmission) or between neighbouring territories (inter-group transmission), where a neighbour was a contiguous territory either directly to the north, east, south or west of the focus territory. Data for intra- and inter-group infections were derived from studies using proximity data loggers and thus do not represent records of actual transmission events. As a consequence, transmission *via* these routes may be overestimated in the model, although there are no data on transmission events within free-living badger populations that can be used to quantify the extent of any overestimation. The structure of the model allowed inter-group transmission only with immediate neighbours or *via* dispersal, although inter-group contacts with non-neighbours were occasionally recorded *via* the field data. However, since inter-group contacts are rare and were not identified as important within the sensitivity analysis, this impact of this minor difference on the results of the model is likely to have been minimal.

**Table 1:** The parameters originally used in the model

**Parameters**			**Values**	**References**
Disease progression	Male	Latent to Infectious	0.297	[15]
		Infectious to latent	0.149	
	Female	Latent to Infectious	0.248	
		Infectious to latent	0.539	
Colonisation	Adult	Male	0.025	[55]
	Adult	Female	0.025	
Dispersal	Adult	Male	0.06	[15,55]
	Adult	Female	0.02	
Fecundity		Coeff A	0.6	[56]
		Coeff B	0.82	
Natural mortality (μ)	Adult	Male	0.304	[10,57]
		Female	0.236	
	Yearlings	Male	0.304	
		Female	0.236	
	Cub	Without Female	0.95	
		Min	0.19	
		Max	0.85	
Disease-induced mortality (α)	Adult and Yearling	Male	0.208	[57]
	Adult and Yearling	Female	0.093	
	Cub	Male	0.208	
	Cub	Female	0.093	
External infection	Probability of external transmission	Summer	0.0001-0.01	
Internal infection	Annual intra-group infection transmission		0.175	[25]
	Annual inter-group infection transmission		0.075	

The values are probabilities per individual per season. Values used are the same in each season unless stated.

Data from the simulations were obtained from the central 10 x 10 territories only, in order to avoid biases due to edge effects. Time in the simulation progressed in discrete iterations corresponding to the four seasons in a year of equal duration *i.e.* three months. A second layer to the simulation represented the habitat quality of each territory in the form of a carrying capacity, with the link between this and badger group size being established through density-dependent cub mortality [15]. The balance between fecundity and cub mortality was calibrated in an initial series of runs under a range of habitat qualities (equilibrium group sizes 2 to 12, representing the full range of group sizes recorded in Europe), to establish the parameter values that maintained the required equilibrium group sizes. Assumptions within the model were as follows: only adult females can breed; breeding is unaffected by disease status; dispersal is by adults and to contiguous territories only; all cubs are born susceptible to disease; there is no vertical or pseudo-vertical disease transmission; and there is homogeneous mixing within social groups. The impact of culling was not explored in these scenarios.

### Sensitivity analysis

We used boosted regression trees to determine which parameters had the greatest influence on badger group size and disease prevalence for group sizes 4, 8 and 12. These analyses were carried out using the ‘R’ statistical and programming environment (R 2.7.0, R Development Core Team 2010).

### Modelling the different scenarios

Initially, we ran the model across a range of equilibrium group sizes (2 to 12 individuals per group in increments of 2), with the initial equilibrium group size being identical across the grid at the start of each simulation. The model was run for 50 years (200 seasons) to allow the populations to stabilise according to the pre-determined equilibrium group size [15]. Then, we introduced infection in different ways as described below and allowed 25 years for the disease dynamics to stabilise. Data were then recorded for the following 50 years. Each model configuration was run 50 times. We recorded the following data every ‘summer’ season: group size; number of empty/filled territories (grid cells); number of intra-group infections; number of inter-group infections; number of external infections; and number of infectious groups.

For scenario 1 (solely badger-derived infection), bTB was introduced in the form of a single infected badger in a single group, selected at random. For scenario 2 (badger-derived infection, supplemented by trickle-type infection from an external source), bTB was introduced as in scenario 1, but then supplemented by trickle-type infection across the whole grid on an annual basis (once each year during the summer). Summer was chosen as it represents the season with the greatest risk of transmission from external sources, due to cattle returning to pasture in late spring and summer, combined with increases in badger territory sizes and foraging activity in summer [8]. The simulations were run with the probability of external transmission set at 0.0001, 0.001 and 0.01 per season for each susceptible individual.

### Analysis of results

We calculated the means, standard deviations and 95% confidence intervals for prevalence, intra-group infection rate, inter-group infection rate, the number of external infectious contacts, the number of empty territories and the number of infected groups based on the final 50 years of the simulations. Outputs from simulations using lower equilibrium group sizes (2–6) were non-parametrically distributed, although the larger equilibrium group sizes yielded parametric data.

## Competing interest

The authors declare that they have no competing interests.

## Authors’ contributions

PCLW, MRH, GM and JH conceived and designed the study. JH and MTB conducted the modelling and the sensitivity analysis. All authors participated in drafting the manuscript. All authors have read and approved the final manuscript.

## Supplementary Material

Additional file 1 Sensitivity analysis of model. Click here for file
