# Supplementary material for: Impact of external sources of infection on the dynamics of bovine tuberculosis in modelled badger populations
Source: BMC Vet Res. 2012 Jun 27;8:92. doi: 10.1186/1746-6148-8-92 (PMC3503836; doi:10.1186/1746-6148-8-92)

**Impact of external sources of infection on the dynamics of bovine tuberculosis in modelled badger populations**

**Supplementary Information: Sensitivity analysis of model**

**Methods**

We carried out sensitivity analysis to determine the influence of different independent parameters (Table A1) on mean badger group size and prevalence. For each parameter, a range of values was obtained by using a Latin-hypercube simulation approach [1]. This involved randomly selecting values from a uniform probability distribution between defined minimum and maximum values. The mean (default) values were based on the available literature. The maximum and minimum values were calculated as 40% and 160%, respectively, of the mean values derived from the literature, following the approach of Shirley et al. [1] and chosen to represent plausible but sufficiently wide spans of values. Determining the maximum and minimum values directly from the literature was not possible due to the lack of available data.

Two hundred randomly-generated combinations of parameter values (simulation configurations) were run in the model 50 times. Simulation configurations consisted of all possible combinations of the model scenarios with the three external infection probabilities (0.0001, 0.001 and 0.1) and for three equilibrium group sizes (4, 8 and 12). Each simulation run consisted of 200 iterations (50 years) to stabilise the model, with values for analysis recorded for the subsequent 200 iterations.

We used regression analysis to identify which model parameters had the greatest influence on the dependent simulation variables (prevalence and mean group size). As values for the simulation parameters within the Latin-hypercube process were chosen from independent distributions, values selected for these parameters were orthogonal to each other and therefore colinearity was not an issue. However, data exploration revealed complex non-linear patterns, indicating that standard linear regression analyses would not be appropriate.

To cope with the complexities in the structure in the data we used a boosted regression tree (BRT) approach [2] using the gbm package [3] in the R statistical software (R Development Core Team 2011), and supplementary functions provided by Elith and Leathwick [4]. BRTs allow for the calculation of relative influence for each independent variable upon the dependent variable. This method can utilise both non-parametric and linear data more easily than generalised linear models and has the advantage of not requiring the a priori definition of interaction terms to be included; these are identified as part of the tree building process. The method utilises decision trees to partition regions in independent variable space resulting in similar values for the dependent variable. The boosted part comes from the repeated development of further trees on earlier trees based on model fitting to the residuals of the previous tree structure. For these analyses we used a tree complexity of 5 (i.e. allowing for up to 5-way interaction terms), learning rate (how quickly the method should converge on a solution) of 0.01 and selected a bag size of 0.5. The method is stochastic and utilises a random subsample of the data to produce each ‘branch’ of the tree, with the remaining data being used for cross-validation. The bag size of 0.5 indicates that at each stage, 50% of the data should be utilised for developing the next ‘branch’ of the tree, and 50% for the cross-validation process. The values used are those recommended for generating good tree structures for a variety of test data sets [3,5]. Cross-validation avoids over-fitting of the model, by repeatedly testing the accuracy of the ‘branches’ that are being ‘built upon/grown’ [5,6]. The estimates from the BRTs were used as measures of the sensitivity of the dependent variables (prevalence and mean group size) to each of the model parameters varied in the sensitivity analysis.

**Results**

*Prevalence*

In the absence of external sources of infection (scenario 1), there were no dominant factors influencing bTB prevalence (Figure A1). Disease-induced mortality, specifically that for adult and yearling females, had the greatest effect on prevalence overall, explaining 8-22% of the variation in prevalence, and this effect was more important for the smaller group sizes. Colonisation was also important in introducing and maintaining disease, although its effect was reasonably consistent across the three equilibrium group sizes, explaining between 12-20% of variation in prevalence.

For the lowest level of external contact (scenario 2), intra-group transmission was the single most important influence on disease prevalence, explaining around 80% of the variation in prevalence. Group size had no impact on the relative importance of the different variables on prevalence. This pattern was also consistent for group sizes of 8 and 12 at the higher levels of external contact; at these group sizes, the level of external contact had little effect on disease. However, for group size 4, as the level of external contact increased, the relative influence of intra-group transmission on prevalence declined, to 60% at an external contact level of 0.001 and 40% at an external contact level of 0.1. As the influence of intra-group transmission declined, other disease-related factors became more important, particularly adult and yearling female disease-induced mortality and the balance of transfer between latent and infectious states.

*Mean group size*

In the absence of external infectious contact (scenario 1), there were no dominant factors influencing group size (Figure A2). With the lowest rate of external contact (scenario 2), colonisation and dispersal had the greatest influence on group size, accounting for over 97% of the variation in group size for equilibrium group sizes of 4 and 8. As the probability of external infection increased, the influence of colonisation and dispersal on group size declined slightly, and disease-related parameters, specifically the intra-group transmission probability and the probability of transfer between latent and infectious states, also became important in influencing group size, accounting for up to 30% and 40% respectively of variation in group size. Female population and disease parameters had a greater influence on group size than male ones, reflecting the dependence of groups on females being present to produce cubs and reduce the likelihood of stochastic die-offs.

**Conclusions**

Colonisation and dispersal, especially of adult females, were important in influencing mean group size in the absence of external infection. The smaller the group, the relatively greater the influence of the number of females, since this directly affects the group’s reproductive potential. As group size increases, a group is more likely to contain more females, and hence the relative importance of the female parameters declines. As rates of external infection increase, colonisation becomes less important in influencing group size, and disease-related parameters such as intra-group transmission and the rate of transfer between infectious and latent states assume a greater importance.

Intra-group transmission was the dominant disease-related parameter overall in terms of its effect on prevalence. This reflects the spatio-temporally persistent nature of bTB in badger populations, and is representative of a disease that is generally maintained through interactions within rather than between groups. At lower group sizes, where the disease is at or below the threshold population density predicted by the model, the parameters of infection itself, such as disease-induced mortality and the rates of transfer between different infectious states, become more important in influencing prevalence.

**References**

1. Shirley MDF, Rushton SP, Smith GC, South AB, Lurz PWW: **Investigating the spatial dynamics of bovine tuberculosis in badger populations: evaluating an individual-based simulation models**. *Ecol Model* 2003, **167**:139-157.
2. Leathwick JR., Elith J, Francis MP, Hastie T, Taylor P: **Variation in demersal fish species richness in the oceans surrounding New Zealand: an analysis using boosted regression trees.** *Mar Ecol Prog Ser* 2006*,* **321**:267-281.
3. Ridgeway G: Generalized Boosted Models: A guide to the GBM package. [www.cran.r-project.org](http://www.cran.r-project.org/), 2007, accessed 16^th^ February, 2011.
4. Elith J, Leathwick JR: **Appendix 3: on-line tutorial on BRTs**. *J Anim Ecol 2008,* **77:**802-813. [*http://onlinelibrary.wiley.com/doi/10.1111/j.1365-2656.2008.01390.x/suppinfo*](http://onlinelibrary.wiley.com/doi/10.1111/j.1365-2656.2008.01390.x/suppinfo), accessed 14^th^ February, 2011.
5. Elith J, Leathwick JR, Hastie T: **A working guide to boosted regression trees**. *J Anim Ecol* 2008, **77**:802-813.
6. Elith J, Graham CH, Anderson RP, Dudik M, Ferier S, Guisan A, Hijmans RJ, Huettmann F, Leathwick JR, Lehman A, Li J, Lohmann LG, Loiselle BA, Manion G, Moritz C, Nakamura M, Nakazawa Y, Overton JMM, Townsend Petersen A, Phillips SJ, Richardson K, Scachetti-Pereira R, Schapire RE, Soberon J, Williams S, Wisz MS, Zimmermann NE: **Novel methods improve prediction of species’ distributions from occurrence data**. *Ecography* 2006, **29**:129-151.
7. Woodroffe R, Macdonald DW, da Silva J: **Dispersal and philopatry in the European badger, *Meles meles***. *J Zool* 1995, **237**:227-239.
8. White PCL, Harris S: **Bovine tuberculosis in badger (*Meles meles*) populations in south-west England: the use of a spatial stochastic simulation model to understand the dynamics of the disease**. *Phil Trans R Soc Lond B* 1995, **349**:391-413.
9. Wilkinson D, Smith GC, Delahay RJ, Rogers LM, Cheeseman CL, Clifton-Hadley RS: **The effects of bovine tuberculosis (*Mycobacterium bovis*) on mortality in a badger (*Meles meles*) population in England**. *J Zool* 2000, **250**:389-395.
10. Böhm M, Palphramand KL, Newton-Cross G, Hutchings MR, White PCL: **The spatial distribution of badgers, setts and latrines: the risk for intra-specific and badger-livestock disease transmission**. *Ecography* 2008, **31**:525-537.

Table A1. Range of parameter values used in the sensitivity analysis

| **Parameter** | **Estimated value (default value)** | **Minimum value** | **Maximum value** | **Literature used** |
| --- | --- | --- | --- | --- |
| Colonisation |  |  |  |  |
| Annual probability for adult male | 0.025 | 0.01 | 0.04 | [7] |
| Annual probability for adult female | 0.025 | 0.01 | 0.04 | [7] |
| Dispersal |  |  |  |  |
| Annual probability for adult male | 0.060 | 0.024 | 0.096 | [8] |
| Annual probability for adult female | 0.020 | 0.008 | 0.032 | [8] |
| Disease-induced mortality | | | | |
| Adult and yearling males | 0.208 | 0.112 | 0.3328 | [9] |
| Adult and yearling female | 0.093 | 0.0372 | 0.1488 | [9] |
| Cubs – male | 0.208 | 0.112 | 0.3328 | [9] |
| Cubs – female | 0.093 | 0.0372 | 0.1488 | [9] |
|  |  |  |  |  |
|  |  |  |  |  |
| Disease dynamics  Annual intra-group bTB transmission | 0.175 | 0.07 | 0.28 | [10] |
| Annual inter-group bTB transmission | 0.0075 | 0.003 | 0.012 | [10] |
|  | | | | |
| Male infectious to latent | 0.149 | 0.0596 | 0.2384 | [8] |
| Male latent to infectious | 0.297 | 0.1188 | 0.4752 | [8] |
| Female infectious to latent | 0.539 | 0.2156 | 0.8624 | [8] |
| Female latent to infectious | 0.248 | 0.0992 | 0.3968 | [8] |

Figure A1. Stacked column chart showing the percentage influence of each independent variable on prevalence for a given scenario (s), group size and external transmission probability.


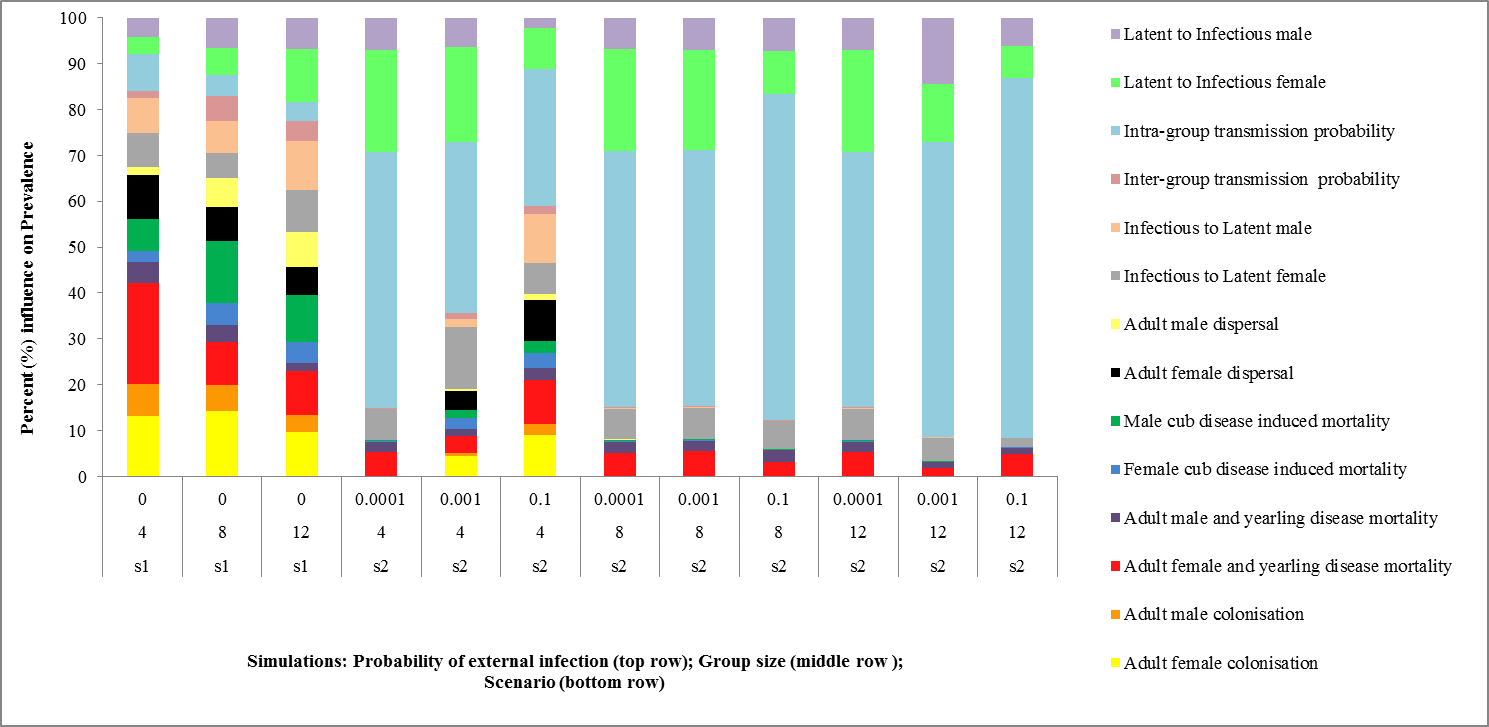


Figure A2. Stacked column chart showing the percentage influence of each independent variable on mean group size for a given scenario (s), group size and external transmission probability


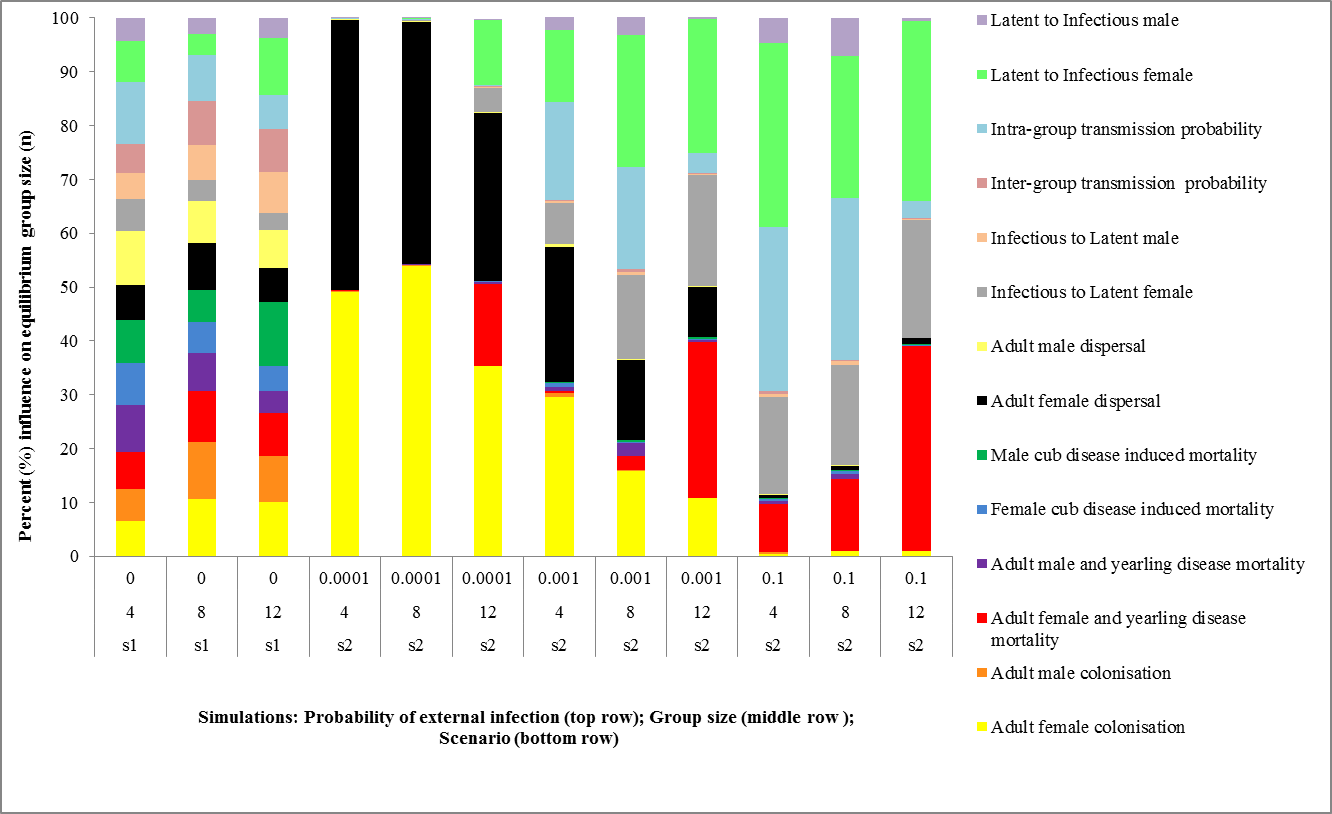

Supplement: Additional file 1 — Sensitivity analysis of model. [file 1746-6148-8-92-S1.doc]
